# Analysis of Blood Concentrations of Zinc, Germanium, and Lead and Relevant Environmental Factors in a Population Sample from Shandong Province, China

**DOI:** 10.3390/ijerph14030227

**Published:** 2017-02-24

**Authors:** Long Li, Guang Xu, Hua Shao, Zhi-Hu Zhang, Xing-Fu Pan, Jin-Ye Li

**Affiliations:** 1 School of Medicine and Life Sciences, University of Jinan-Shandong Academy of Medical Sciences, Jinan 250062, China; tianya584526@163.com; 2 Shandong Academy Occupational Health and Occupational Medicine, 18877 Jingshi Road, Jinan 250062, China; sunshinefeng123@163.com; 3 Department of Occupational Diseases Control and Prevention, Fengtai Center for Disease Control and Prevention, Beijing 100071, China; cengcengc3@163.com; 4 Shandong Provincial Western Hospital, Jinan 250022, China; lijinyejn@163.com

**Keywords:** reference values, blood, metal, trace element

## Abstract

Trace elements, including zinc (Zn) and germanium (Ge), are essential for health; deficiency or excess levels of trace elements results is harmful. As a result of industrial and agricultural production, Pb widely exists in people’s living environment. It is absorbed mainly through the respiratory and digestive tracts, producing systemic harm. Reference values for a normal, healthy population are necessary for health assessment, prevention and treatment of related diseases, and evaluation of occupational exposures. Reference ranges for the Chinese population have not been established. From March 2009 to February 2010; we collected data and blood samples (*n* = 1302) from residents aged 6–60 years living in Shandong Province, China. We measured blood concentrations of Zn, Ge, and Pb using inductively coupled plasma mass spectrometry to determine reference ranges. Results were stratified by factors likely to affect the concentrations of these trace elements: sex, use of cosmetics or hair dye, age, alcohol intake, smoking habits, and consumption of fried food. The overall geometric mean (GM) concentrations (95% confidence interval) were 3.14 (3.08–3.20) mg/L for Zn, 19.9 (19.3–20.6) μg/L for Ge, and 24.1 (23.2–25.1) μg/L for Pb. Blood Zn concentrations were higher in women than in men (*p* < 0.001), while the opposite was found for Pb (*p* < 0.001) and sex did not influence Ge (*p* = 0.095). Alcohol use was associated with higher blood concentrations of Zn (*p* = 0.002), Ge (*p* = 0.002), and Pb (*p* = 0.001). The GM concentration of Zn was highest in 20–30-year-olds (*p* < 0.001), while Pb concentrations were highest in 12–16-year-olds (*p* < 0.001). Use of hair dye was associated with lower blood concentrations of Ge (*p* < 0.05). GM blood concentrations of Pb differed significantly between those who consumed fried foods 1–2 times/month (18.7 μg/L), 1–2 times/week (20.9 μg/L), and every day (28.5 μg/L; *p* < 0.001). Blood Pb concentrations were higher in subjects who used cosmetics (*p* < 0.05), hair dye (*p* < 0.05), and who smoked cigarettes (*p* < 0.001) than in those who did not.

## 1. Introduction

The development of mining and manufacturing industries has led to a rise in occupational and non- occupational metal poisoning, which has become a major public health problem. To assess and monitor risk, reference values of potentially harmful metals-including trace elements required by the body-for a normal, healthy population are essential for comparison purposes [1,2]. Reference values enable assessment of population health, disease prevention and treatment [3], and evaluation of occupational exposures and environmental conditions [4].

Trace elements are essential to the human body, even though the daily demand for them is very low. They are involved in bodily physiology [5,6,7] and are important components of vitamins, hormones, and enzyme systems [8,9]. Insufficient or excess trace element concentrations in the body will result in harm [10].

The normal human body contains 2–4 g of zinc (Zn), one of the fourteen essential trace elements [11,12]. Biologically, Zn has three main functions: catalysis, adjustment, and structure. Zn, an enzyme constituent [11,13], also catalyzes enzymatic reactions and plays an important role in metabolism [14,15], tissue respiration [16,17] and regeneration [18,19], and growth [17,20]. Zn may also affect the effectiveness of hormone receptor and target organ responses; hormone production, storage, and secretion; and sexual development [21,22]. Zn stabilizes insulin structure [23,24], maintains normal dark adaptation capacity [25,26], and is a component of salivary proteins that promote the sense of taste and appetite [27,28]. It is involved in iron transport and transfer [29,30,31] and enhances the immune system [32,33,34]. Zn can inhibit lipid peroxidation and thiol oxidation of biomembranes, and along with copper-protein, catalase, and vitamin E, maintains cell structure [35,36,37,38]. Zn deficiency causes digestive disorders, delayed sexual maturity/development of secondary sexual characteristics resulting in stunted growth, skin disease, stomatitis, alopecia [29,30,39], and can impair immune function. Zn overdoses can cause metal fume fever, reduce serum high-density lipoprotein-cholesterol levels, and lead to iron deficiency anemia and copper deficiency [40,41].

Germanium (Ge), another essential trace element, is widely distributed in the body. It is a constituent of the amino acid guanidine and the enzymes cytochrome oxidase and carbonic anhydrase. It is also distributed in the brain cortex and is a component of cell walls, chromosomes, vesicles, lysosomes, and cytoplasmic matrix. Ge has anti-mutation, anti-cancer [42,43], anti-aging [44,45], anti-malarial [46,47], and anti–inflammatory [48,49] properties. It can stimulate hematopoiesis [50] and enhance immune function [51,52,53]. Intake is through consumption of water, food, or drugs [54]. Excessive Ge can damage the kidneys [48,55,56], nervous system [57,58,59], and lungs [60].

Lead (Pb), widely distributed in the atmosphere, soil, and food, enters the body via the respiratory and digestive systems and is deposited in bones. Unlike Zn and Ge, Pb is not beneficial to health. Pb can result in neurologic [61,62,63], skeletal, reproductive [64,65], hematopoietic [66,67], and urinary system [68,69] toxicity.

Considering their beneficial (Zn and Ge) and harmful (Pb) effects, periodic biological monitoring of these trace elements should be conducted. In Europe and the USA, such monitoring is generally performed at the national level. However, in Shandong Province, China, no previous biomonitoring studies have been conducted, and it is not known whether the geographical or social characteristics of this area make it nationally representative. Therefore, we aimed to measure the blood ^66^Zn, ^72^Ge, and ^208^Pb concentrations of the general population in Shandong Province, stratified by sex, age, smoking, alcohol consumption, cosmetic and hair dye use, and fried food consumption, to form a basis for biological monitoring and scientific research.

## 2. Materials and Methods

### 2.1. Subject Selection

**Figure 1:**
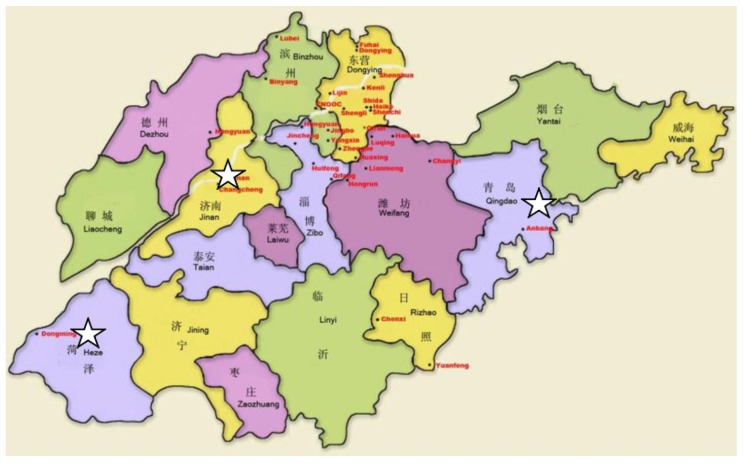
Location of the study population. Shandong Province, located in the east of China, has a population of 92.8 million people and a land area of 153,300 km^2^. We included 418 research participants from Qingdao, 345 from Jinan, and 539 from Heze. ☆: Sample collection areas.

Shandong Province, located in the east of China, has a population of 92.82 million people and a surface area of 153,300 km^2^. Using cluster sampling, the study subjects were selected as follows: First, Shandong Province was divided into three socioeconomic levels. Second, we randomly sampled one city from each socioeconomic level. Last, we randomly selected 1302 subjects in each community. Inclusion criteria were as follows: (1) living in the local area for at least five years; (2) living in areas without relevant industrial pollution; (3) no history of liver or kidney diseases, diabetes, hyperthyroidism, cancer, or other chronic diseases; (4) no acute infection; (5) no use of pharmaceutical preparations or dietary supplements containing trace elements within the past 3 months; and (6) age 6–60 years old. Selected areas are shown in Figure 1: 418 research subjects from Qingdao were included, 345 from Jinan, and 539 from Heze.

All participants completed a questionnaire regarding personal information, lifestyle and eating habits, and medical history. All questionnaires were recovered and meet the requirements. A total of 1302 blood samples and questionnaires were collected from March 2009 to February 2010. Written informed consent was obtained from each subject. The study was conducted in accordance with the Declaration of Helsinki, and the study was approved by the Ethical Censorship Committee of the Shandong Academy of Medical Sciences (YKYLI-2009066). Participants agreed to the use of their blood samples for this biological monitoring research.

### 2.2. Sample Preparation and Analysis

All samples were collected and processed in a clean environment. Blood samples (6 mL) were collected in vacutainers containing lithium heparin (BD, Bergen, NJ, USA), and were immediately transferred to 2 mL freezing tubes (Axygen, San Francisco, CA, USA) after thorough mixing. All samples were stored at −80 °C until analysis. Before analyzing, the samples were warmed to room temperature (23 °C). As described in previous literature [70], 0.5 mL of blood was added to 4.5 mL of a diluent containing 0.01% (V/V) Triton-X-100 (Sigma Aldrich, Bergen, NJ, USA) and 0.5% ultrapure concentrated nitric acid (Merck, Darmstadt, Germany). Samples were vortexed in a table-top vortexer (Multi Reax [XWT-204], Heidolph, San Francisco, CA, USA). Concentrations of Zn, Ge, and Pb in the diluted samples were then quantified using inductively coupled plasma mass spectrometry (ICP-MS, Thermo Fisher, Waltham, MA, USA). Yttrium (Y) solution with concentration of 10 μg/L was used as the internal standard. The 0.01% Triton-X-100 and 0.5% ultrapure by 10 determination will respond to signals corresponding to 3 times the standard deviation of the analyte concentration as the detection limit; the 10 times value was determined by a solvent blank, and the response signals corresponding to 10 times the standard deviation of the measured element concentrations were defined as a quantification limit. Since the method for sample processing calls for dilution by a factor of 10, the limit of detection and limit of quantification were both multiplied by 10. This resulted in limits of detection for Zn, Ge, and Pb of 4.30, 0.18 and 0.28 μg/L, respectively.

### 2.3. Quality Control

Contamination in the pre-analytic phase during sample collection may lead to inaccurate measurements [71]. Therefore, to minimize contamination, we pre-tested the heparin vacutainers and frozen vials. We soaked 20 vacutainers and 20 vials in 1% (V/V) ultrapure nitric acid for one hour and then determined the metal concentrations in the soaking solution using ICP-MS. The concentrations of Zn, Pb, and Ge in these vacutainers and vials were lower than the respective detection limits. Sets of 30 samples were processed after determination of a single point standard solution, provided that the determination result was within the allowable range (deviation < 10%) using nickel, arsenic, molybdenum, and tungsten as reference elements. The ICP-MS measurement procedures were referenced to previous research [72]. Sample preparation and analysis were performed by investigators with professional training and ICP-MS operators with professional experience in occupational hygiene and chemical analysis. The quality of laboratory instruments and procedures was periodically checked to ensure the reproducibility and recovery of the assays; using spiked recovery experiments, recovery was in the range of 90.0% (Pb) to 112.4% (Zn).

### 2.4. Statistical Analysis

All analyses were performed using SPSS version 22.0 statistical package (SPSS, IBM, Chicago, IL, USA) and EpiData 3.1 (EpiData ISOC, Funen, Denmark). The construct validity of the scale was evaluated by principal component analysis (PCA). The distributions of continuous variables were shown by the Kolmogorov-Smirnov test to be non-normal. Therefore, metal concentrations were described in terms of the median and interquartile range (IQR), geometric mean (GM), and 95% confidence interval (95% CI) of the geometric mean. Univariate statistical analysis was performed using the rank sum test. Univariate statistical analyses of the effect of cosmetics, sex, alcohol intake, and hair dye on serum concentrations of trace elements were performed using the Wilcoxon test; the Kruskal-Wallis test was used to assess the effects of age, smoking, and consumption of fried foods on serum concentrations of trace elements. A *p*-value (*p*) ≤ 0.05 was considered statistically significant.

## 3. Results

**Figure 2:**
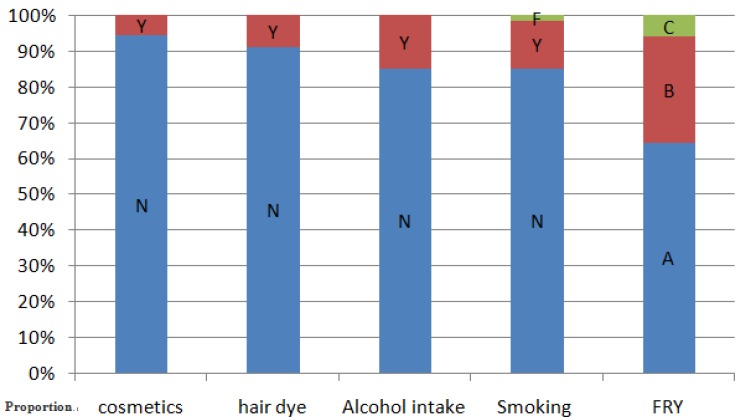
Characteristics of the study population. Univariate analyses of associations between cosmetics, alcohol intake and hair dye with serum concentrations of trace elements were performed using the Wilcoxon test; the Kruskal-Wallis test was used to assess associations between smoking, and consumption of fried foods with serum concentrations of trace elements. N: no; Y: yes; F: former smoker; A: 1–2 times per month; B: 1–2 times per week; C: Every day.

The Kaiser-Meyer-Olkin measure (KMO) value was 0.911, and the partial correlation is very weak; the Bartlett spherical test, rejected the original hypothesis of the unit correlation matrix (*p* = 0.0008), is suitable for factor analysis. There are seven factors which characteristic value in the principal component analysis was greater than 1, and the cumulative contribution to the total variance of the rate of 73.2%. According to the maximum factor load corresponding to the original variables, the original variables are divided into seven categories, which are in good agreement with the seven factors of the scale design. Subjects (765 men and 537 women) were grouped by age as follows: 6–12 (*n* = 231), 12–16 (*n* = 214), 16–20 (*n* = 168), 20–30 (*n* = 187), 30–45 (*n* = 255), and 45–60 (*n* = 247) years. The utilization rate of cosmetics, hair dye and alcohol were 5.5%, 8.6% and 14.7%, respectively. Other demographic data are presented in Figure 2. Chemical concentrations were above the limit of detection for all three trace elements. As shown in Table 1, the GM concentration of blood Zn (BZn) was 3.14 mg/L (95% CI: 3.08–3.20 mg/L). Women had significantly higher levels of Zn than men (GM, 3.28 mg/L vs. 3.04 mg/L, *p* < 0.001). Alcohol use was associated with slightly higher BZn concentrations (GM for drinkers = 3.39 mg/L; GM for non-drinkers = 3.09 mg/L; *p* = 0.002). The GM concentration of BZn was highest in the 20–30-year-old age group (*p* < 0.001).

The GM concentration of blood Ge (BGe) for the total sample was 19.9 μg/L (95% CI: 19.3–20.6 μg/L), as shown in Table 2. The GM of BGe among the subjects who used hair dye was 17.6 μg/L, which was significantly lower than that of non-users (GM = 20.2 μg/L, *p* < 0.05). The GM concentration of BGe in the 16–20-year-old age group was significantly higher than in the other age groups (*p* < 0.001). Alcohol consumption was associated with slightly increased BGe levels (GM for drinkers = 19.6 μg/L; GM for non-drinkers = 21.7 μg/L; *p* = 0.002). A statistically significant difference in blood Ge concentration was observed according to consumption of fried foods: 1–2 times per month (GM = 18.7 μg/L), 1–2 times per week (GM = 20.9 μg/L), or every day (GM =28.5 μg/L), *p* < 0.001.

The geometric mean blood concentration of Pb (BPb) was 24.1 (95% CI: 23.2–25.1 μg/L) (Table 3). Men had higher BPb (GM = 26.6 μg/L, 95% CI: 25.3–27.9 μg/L) than women (GM = 20.6 μg/L, 95% CI: 19.34–21.8 μg/L, *p* < 0.001). The GM concentration of BPb was significantly higher in subjects who used cosmetics compared to those who did not (24.4 μg/L vs. 20.0 μg/L, *p* < 0.05), and was also higher in subjects who did than did not use hair dye compared to those who did (24.5 μg/L vs. 19.6 μg/L, *p* < 0.05). Alcohol intake was associated with elevated BPb (GM for drinkers = 28.3 μg/L; GM for non-drinkers = 23.4 μg/L; *p* = 0.002). The GM concentration of BPb was highest in the 12–16-year-old age group (*p* < 0.001). There was a statistically significant difference in BPb between smokers (GM = 29.1 μg/L, 95% CI: 26.3–32.0 μg/L) and non-smokers (GM = 23.1 μg/L, 95% CI: 22.1–24.1 μg/L, *p* < 0.001).

## 4. Discussion

Zn, Ge, and Pb are present in food, water, soil, and elsewhere in the natural environment. Appropriate levels of trace elements are required to maintain the body healthy. Therefore, knowing the reference ranges for these metals is useful for evaluation of occupational hazard exposure and to evaluate prevention or treatment strategies for diseases caused by deficiency or excess of these elements. Our results show that levels of trace elements in the body were associated with dietary and environment factors. Reference value of trace elements in human blood have been measured in many areas including Europe and North America since the 1990s [2,37]. Blood concentrations reflect short-term changes [73] and are considered a sensitive indicator of trace element deficiency or excess. In the present study, concentrations of Zn, Ge, and Pb were measured in blood, as a reflection of the total body content of these trace elements.

As early as the 1990s, European and American countries began biological monitoring of Zn [74,75]. The reference range for Zn obtained in an Italian sample (GM = 6.42 mg/L) was similar to the ranges obtained in populations in Spain and in the Czech Republic [76,77,78,79,80], but was significantly higher than that shown in our data (GM = 3.14 mg/L). Another study from China found results similar to ours [78]. Rice is the main staple of Asians, but in current high-yielding rice varieties the supply of zinc is poor, as polishing and shelling cause a huge loss of zinc [81,82]. Other reason is possible that discrepancies in levels of trace elements between different countries are partly due to environmental factors, but specific reasons need to be explored further.

The BZn of drinkers was slightly higher than that of non-drinkers. This finding is consistent with results from a study in Italy [76] and likely reflects a causal relationship. Alcohol contains a large amount of Zn, derived from the soil via absorption by plants, which is then released when beverages are packaged in metal containers [83,84]. The GM concentration of BZn was significantly higher in the 20–30-year-old age group than in any other age group studied. This may be because sexual development in this period requires an increased intake of Zn [21,22].

Biological monitoring of Ge is necessary, as this element is used in many drugs. To date, there has been no estimate of Ge concentration from a national sample. Results reported from Chengde City (18.3–92.5 µg/L) [85] were similar to those from our study (GM = 19.9 μg/L), whereas Ge serum levels of 290 µg/L have been reported in the USA [86]. The results of the present study showed that the BGe among the subjects who used hair dye was significantly lower than among those who did not. This may be because hair dye contains a high concentration of Pb, which inhibits the absorption of Ge, but the specific mechanism needs further study. As adolescence is a critical period of growth and development, the demand for trace elements in this period is greatly increased. Accordingly, the BGe in the 16–20-year old age group was significantly higher than in other age groups. Those who consumed alcohol had significantly higher blood Ge concentrations than non-drinkers, and that the greater the frequency of fried food consumption, the higher the level of blood Ge. The reasons underlying these findings require further study.

Because Pb is widespread and harmful biomonitoring studies have been conducted in many countries [87,88,89,90]. Data from the present study revealed GM concentration of BPb to be 24.1 μg/L, which is lower than that observed in Brazilian, Czech, Danish, Italian, and Spanish studies, but higher than in that in American, Korean, Canadian, and Australian studies [79]. There are reports that BPb is higher in men than in women, as was found in our study [77,78]. We found that BPb concentrations were significantly higher in subjects who used cosmetics than in those who did not, similar to the findings for hair dye users. This is consistent with the results of previous reports; the vast majority of cosmetics contain Pb, which is absorbed through the skin into the body [91,92,93,94,95,96]. In the present study, alcohol consumption was associated with higher BPb. Pb in the soil is absorbed into plants [97], and ingestion of alcohol facilitates the absorption of Pb by the body [98]. There was a statistically significant difference in BPb concentrations between smokers and non-smokers. Pb in cigarettes enters the body through the respiratory tract [99], and may act synergistically with risk factors associated with hypertension [100]. Existing literature supports an identical trend with drinkers and smokers [101].

## 5. Conclusions

This study provides data on blood concentrations of Zn, Pb, and Ge in a sample of the population living in Shandong Province, China. It provides valid and reliable reference data for establishing reference values for blood levels of these trace elements for the Chinese population. Alcohol consumption was associated with blood concentrations of Zn, Ge, and Pb, while cigarette smoking had no significant influence on BZn and BPb. Use of cosmetics and hair dye was associated with higher blood Pb concentrations. In addition, there was a positive association between the frequency of fried food consumption and blood Pb concentration. Further research is needed to determine the factors underlying the associations we observed between these variables and blood levels of trace elements.

## Figures and Tables

**Table 1:** Blood ^66^Zn levels (mg/L), stratified by relevant categorical variables.

Items	*n*	P5	P25	P50	P75	P95	GM (95% CI)
Total population	1302	1.41	2.40	3.10	3.83	5.02	3.14 (3.08–3.20)
Sex (*p* < 0.001)							
Male	765	1.32	2.29	3.00	3.74	4.86	3.04 (2.97–3.12)
Female	537	1.55	2.51	3.26	3.99	5.13	3.28 (3.18–3.37)
Use of cosmetics (*p* = 0.991)							
No	1230	1.43	2.40	3.10	3.82	5.01	3.14 (3.08–3.20)
Yes	72	1.23	2.35	3.08	3.97	5.35	3.14 (2.88–3.41)
Use of hair dye (*p* = 0.068)							
No	1190	1.43	2.41	3.13	3.84	5.03	3.16 (3.09–3.22)
Yes	112	1.36	2.25	2.84	3.71	4.92	2.95 (2.76–3.14)
Age in years (*p* < 0.001)							
6–12	231	1.03	1.94	2.72	3.55	4.32	2.75 (2.61–2.88)
12–16	214	1.65	2.49	3.17	3.70	4.91	3.13 (3.00–3.26)
16–20	168	1.86	2.59	3.27	4.33	5.34	3.41 (3.24–3.58)
20–30	187	1.78	2.89	3.63	4.27	5.56	3.61 (3.45–3.77)
30–45	255	1.39	2.22	2.83	3.69	5.08	3.01 (2.88–3.15)
>45	247	1.52	2.38	3.05	3.81	4.77	3.10 (2.97–3.22)
Alcohol intake (*p* = 0.002)							
No	1110	1.40	2.39	3.08	3.75	4.93	3.09 (3.03–3.16)
Yes	192	1.72	2.50	3.32	4.19	5.54	3.39 (3.22–3.56)
Smoking (*p* = 0.001)							
No	1111	1.36	2.39	3.07	3.77	4.96	3.10 (3.04–3.16)
Yes	170	1.72	2.56	3.45	4.18	5.29	3.42 (3.25–3.59)
Former smoker	21	1.44	2.28	2.73	3.52	5.35	2.91 (2.49–3.34)
Consumption of fried foods (*p* = 0.088)							
1–2 times per month	842	1.40	2.39	3.06	3.76	4.91	3.10 (3.03–3.17)
1–2 times per week	380	1.49	2.43	3.19	3.87	5.19	3.17 (3.06–3.28)
Every day	80	1.46	2.30	3.33	4.48	6.11	3.42 (3.12–3.72)

**Table 2:** Blood ^72^Ge levels (μg/L), stratified by relevant categorical variables.

Items	*n*	P5	P25	P50	P75	P95	GM (95% CI)
Total population	1302	5.10	12.1	18.3	24.5	45.2	19.9 (19.3–20.6)
Sex (*p* = 0.095)							
Male	765	4.40	12.5	18.9	25.2	40.9	19.8 (19.0–20.6)
Female	537	6.13	11.9	17.0	23.0	53.0	20.2 (19.1–21.3)
Use of cosmetics (*p* = 0.544)							
No	1230	5.11	12.2	18.3	24.6	43.3	19.9 (19.3–20.6)
Yes	72	4.65	10.4	17.2	24.1	55.3	20.5 (17.1–23.9)
Use of hair dye (*p =* 0.016)							
No	1190	5.11	12.3	18.5	24.6	46.7	20.2 (19.5–20.8)
Yes	112	4.99	10.9	15.3	22.6	38.7	17.6 (15.7–19.5)
Age in years (*p* < 0.001)							
6–12	231	1.04	9.96	17.9	23.1	27.0	16.2 (15.1–17.3)
12–16	214	6.96	14.3	19.9	25.9	42.8	21.5 (20.0–23.0)
16–20	168	9.61	15.0	22.8	45.2	62.2	29.3 (26.5–32.0)
20–30	187	5.21	10.8	16.0	23.1	32.5	17.3 (16.0–18.5)
30–45	255	4.89	12.6	19.4	26.8	42.5	20.9 (19.5–22.3)
>45	247	6.48	11.1	15.3	20.7	34.0	16.9 (15.8–17.9)
Alcohol intake (*p* = 0.001)							
No	1110	4.88	11.8	17.8	24.0	46.9	19.6 (18.9–20.3)
Yes	192	6.44	13.8	20.8	28.0	41.5	21.7 (20.2–23.2)
Smoking (*p* = 0.148)							
No	1111	4.91	11.9	17.9	24.5	47.1	19.9 (19.2–20.6)
Yes	170	5.70	13.8	18.9	24.6	36.4	19.9 (18.5–21.3)
Former smoker	21	10.7	16.1	19.7	25.6	42.0	21.8 (17.9–25.6)
Consumption of fried foods (*p* < 0.001)							
1–2 times per month	842	4.92	11.9	17.6	23.9	37.6	18.7 (18.0–19.4)
1–2 times per week	380	4.93	12.2	18.6	24.9	53.1	20.9 (19.5–22.3)
Every day	80	7.31	16.1	23.6	40.4	63.4	28.5 (24.7–32.3)

**Table 3:** Blood ^208^Pb levels (μg/L), stratified by relevant categorical variables.

Items	*n*	P5	P25	P50	P75	P95	GM (95% CI)
Total population	1302	3.00	12.5	20.9	32.2	56.0	24.1 (23.2–25.1)
Sex (*p* < 0.001)							
Male	765	4.48	14.0	22.9	35.2	58.9	26.6 (25.3–27.9)
Female	537	2.05	10.3	17.9	28.4	47.6	20.6 (19.3–21.8)
Use of cosmetics (*p* = 0.019)							
No	1230	1.41	6.86	17.8	28.8	53.8	20.0 (16.0–24.0)
Yes	72	3.69	12.7	21.1	32.4	56.3	24.4 (23.4–25.3)
Use of hair dye (*p* = 0.004)							
No	1190	0.12	9.12	17.9	27.1	49.0	19.6 (17.0–22.3)
Yes	112	3.66	12.7	21.2	32.8	56.4	24.5 (23.5–25.5)
Age in years (*p* < 0.001)							
6–12	231	1.80	10.8	21.2	36.1	56.9	25.0 (22.7–27.3)
12–16	214	7.48	18.0	25.6	35.6	53.4	27.7 (25.7–29.6)
16–20	168	4.96	11.2	19.3	28.0	57.5	23.2 (20.4–26.0)
20–30	187	5.33	14.9	22.3	34.3	56.5	26.9 (24.1–29.8)
30–45	255	0.07	7.40	18.0	29.4	56.1	20.8 (18.6–22.9)
>45	247	4.99	12.8	18.6	27.4	55.0	22.13 (20.2–24.0)
Alcohol intake (*p =* 0.001)							
No	1110	2.67	12.0	20.4	31.5	51.6	23.4 (22.4–24.4)
Yes	192	5.89	15.6	23.4	37.7	60.7	28.3 (25.6–31.0)
Smoking (*p* < 0.001)							
No	1111	2.62	11.9	19.9	31.5	51.6	23.1 (22.1–24.1)
Yes	170	5.81	16.7	24.6	36.0	69.3	29.1 (26.3–32.0)
Former smoker	21	5.20	22.0	25.2	42.6	153.0	37.9 (23.3–52.5)
Consumption of fried foods (*p* = 0.319)							
1–2 times per month	842	2.94	12.5	20.6	32.4	56.8	24.1 (22.9–25.2)
1–2 times per week	380	3.56	12.1	21.2	31.6	53.0	23.8 (22.1–21.2)
Every day	80	4.36	14.4	23.1	34.8	57.4	26.0 (22.5–29.5)
